# LncRNA *NEAT1* promotes IL-6 secretion in monocyte-derived dendritic cells via sponging *miR-365a-3p* in systemic lupus erythematosus

**DOI:** 10.1080/15592294.2023.2226492

**Published:** 2023-06-21

**Authors:** Mengmeng Xiang, Yilun Wang, Qian Chen, Jie Wang, Zhanyan Gao, Jun Liang, Jinhua Xu

**Affiliations:** a Department of Dermatology, Huashan Hospital, Fudan University, Shanghai, China; b Shanghai Institute of Dermatology, Shanghai, China

**Keywords:** Systemic lupus erythematosus, inflammation, monocyte-derived dendritic cells, interleukin 6, ceRNA, long non-coding RNA

## Abstract

Increasing evidence has uncovered the essential roles of long noncoding RNAs (lncRNAs) in biological and pathological functions of dendritic cells (DCs) among patients with systemic lupus erythematosus (SLE). However, whether lncRNA nuclear paraspeckle assembly transcript 1 (*NEAT1*) could modulate DCs, especially in the inflammation of SLE, remains largely unknown. Fifteen SLE patients and fifteen age-matched healthy controls were included, and their monocyte-derived dendritic cells (moDCs) were cultured in vitro. Our research identified that the expression of *NEAT1* was significantly increased in moDCs of SLE patients and positively correlated with disease activity. Interleukin 6 (IL-6) from both plasma and secreted supernatants of moDCs was also elevated in the SLE group. In addition, regulation of *NEAT1* in moDCs by transfection could lead to the corresponding change in IL-6 generation. While for *miR-365a-3p*, a micro-RNA that can bind with the 3’ UTR region of *IL6* and *NEAT1*, it may serve as a negative modulator since its overexpression could result in the reduction of IL-6 levels and vice versa. Additionally, the elevation in *NEAT1* expression could increase the secretion of IL-6 by specifically binding to *miR-365a-3p*, reducing the negative modulatory effects of *miR-365a-3p* on the *IL6* target gene, which suggested that elevated *NEAT1* expression could function as the competing endogenous RNA (ceRNA). In conclusion, our findings indicate that *NEAT1* can efficiently sponge *miR-365a-3p* to upregulate expression and secretion of IL-6 in moDCs, suggesting that the *NEAT1/miR-365a-3p/IL6* axis may be involved in the development of SLE disease.

## Introduction

Systemic Lupus Erythematosus (SLE), as a chronic autoimmune disease, has a lot of different manifestations. The prevalence of SLE is higher in women, with a female-to-male ratio of 9:1 [1]. SLE’s aetiology is elusive, however genetic predisposition, as well as environmental factors have been suggested to play a role in the pathogenesis [2]. Currently available treatments, including immunosuppressive drugs and glucocorticoids, still have major limitations as they lack complete curative effects and may cause unexpected and severe side effects. Due to the heterogeneity of SLE, other newly emerging target approaches fail to satisfy all patients with varying severity and characteristics [3,4]. Thus, a personalized treatment option based on the individual’s unique molecule profile is needed.

Dendritic cells (DCs) have been recognized as important participants in the pathogenesis of SLE over the last decade, making them potential therapeutic targets for immune system fine-tuning [5]. DCs go through a complicated process of maturation into antigen-presenting cells and subsequently activate T cells in the response to immunogens, whereas immature DCs transmit self-antigens to lymphocytes without co-stimulation, resulting in peripheral tolerance [6]. In several murine models, it has been found that DCs carrying self-antigens can induce autoimmune disorders [7]. Changes in DC homoeostasis have also been linked to a variety of human autoimmune illnesses, such as type 1 diabetes, rheumatoid arthritis, and SLE [8–10]. DCs and monocyte-derived dendritic cells (moDCs) from SLE patients exhibit significant differences in biological behaviour, for example, moDCs from SLE patients may secrete higher levels of proinflammatory cytokines, and they are characterized by higher expression of maturation and differentiation markers and have enhanced ability to prime T cells [11].

Long non-coding RNAs (LncRNAs) are a kind of newly emerging modulators that have epigenetic regulatory capabilities in various biological or pathologic processes of the immune system [12]. Several lncRNAs, such as lnc-Dpf3 and lnc-DC, have been identified to play a vital role in DC’s maturation, differentiation, and migration [13–15]. LncRNAs can interact with various molecules including DNA, proteins, and other RNA, and they can also alter targeted mRNA expression by sponging microRNAs for the same binding sites, as the ceRNA hypothesis suggests [16]. Several differentially expressed lncRNAs in SLE moDCs were identified from microarray in our previous research, but their functions in the initiation and progression of SLE have not yet been well understood [17]. One of the lncRNAs, nuclear paraspeckle assembly transcript 1 (*NEAT1*), has received considerable attention due to its validated associations with tumour migration and progression [18]. Additionally, it has been shown to play a role in the inflammatory activities of immune cells including macrophages and monocytes [19]. However, few studies have explored its underlying effects in dendritic cells and SLE. This study was performed in order to ascertain if *NEAT1* can influence inflammatory functions of moDCs in SLE and how *NEAT1* may engage in this process.

## Materials and methods

### Patients and healthy controls

Patients diagnosed with SLE according to the 1997 American College of Rheumatology (ACR) revised criteria were enrolled from Huashan Hospital, Fudan University. Disease activity of each patient was evaluated and scored by The Systemic Lupus Erythematosus Disease Activity Index 2000 (SLEDAI-2K). Healthy controls with matched age and sex composition were also enrolled. Blood samples were obtained with informed consent and approval by the Independent Ethics Committee of Huashan Hospital. The experiments were conducted following the pertinent guidelines and rules of Huashan Hospital. Comparisons between moDCs from SLE patients and healthy controls were performed followed by molecular experiments carried out on the moDCs from healthy participants.

### PBMC separation and moDcs culture

Peripheral blood mononuclear cells (PBMC) were separated from whole blood samples of all included SLE patients and healthy controls using the Ficoll gradient centrifugation (GE Healthcare, USA). Then PBMC acquired were separated by positive selection (purity >90%) with CD14^+^ magnetic beads from Miltenyi (German). Sorted CD14^+^ monocytes (1 × 10^6^/mL) were cultured for 5–7 days in the medium consisting of RPMI 1640 added with 10% fetal bovine serum (FBS) and antibiotics (1×penicillin/streptomycin). Medium was supplemented with cytokines containing 60 ng/mL granulocyte-macrophage colony-stimulating factor (GM-CSF) and 60 ng/mL interleukin-4 (IL-4) (R&D, USA) to induce the differentiation towards dendritic cells. On day 5 of culture, transfections were performed on immature moDCs. To stimulate moDCs maturation, 1 ug/mL of LPS (*Escherichia coli* O55:B5; Sigma-Aldrich, USA) was added to the medium on day 6. On day 7 or day 8, matured moDCs were harvested for the following studies.

### Quantitative real-time PCR (Qrt-PCR)

Total RNA from matured moDCs was extracted with TRIzol (Invitrogen, USA) following the manufacturer’s instructions. Prime Script™ RT Master Mix kit was utilized to reversely transcribe cDNAs of mRNA and lncRNA from 0.5 μg total RNAs (Takara, Japan). For microRNA analysed in the study, reverse transcription was completed with Bulge-Loop miRNA qRT-PCR Starter Kit (Ribobio, China). qRT-PCR for all RNA was done with the TB Green™ Premix Ex Taq™ II (Takara, Japan) on an ABI 7500 Fast Real-time PCR System (ABI, USA). The ΔΔCT approach was applied to quantitatively normalize relative expressions of target RNAs to glyceraldehyde 3-phosphate dehydrogenase (GAPDH), beta-actin (ACTB), or U6 expression. Table S1 lists all of the qRT-PCR primers utilized.

### RNA fluorescence in situ hybridization (FISH)

The FISH probe targeting *NEAT1* was designed and synthesized by Ribobio (China). Matured moDCs were permeabilized with 0.5% Triton X-100 for 10 minutes at 4°C after being fixed in 4% paraformaldehyde for 15 minutes at room temperature. Pre-hybridization was completed at 37°C for 30 minutes and then hybridization was done using *NEAT1* probe sets and the RiboTM Fluorescent In Situ Hybridization Kit (Ribobio, China) at 37°C overnight. Considering the intracellular location for 18S is mostly in the cytoplasm and for U6 is the nucleus, 18S, and U6 were chosen as positive controls in FISH.

### Dual-luciferase-reporter gene assay

To validate that *NEAT1* and *IL6* were both direct targets of *miR-365a-3p*, we applied the dual-luciferase-reporter gene assay. *NEAT1* and 3′UTR of *IL6* sequences were cloned into pSI-Check2 plasmids (Hanbio, China). Luciferase constructs carrying wildtype or mutant sequences were transfected into 293 T cells when cell confluence reached 50–70%. *MiR-365a-3p* mimic or negative control (NC) mimic was also added. Forty-eight hours after transfection, the cells were collected and assessed with the dual-luciferase-reporter kit (Promega, USA). Luciferase expression was evaluated by the measurement of luminescence and the activity of firefly luciferase was normalized to that of Renilla luciferase.

### Cell transfection

Down-regulation of *NEAT1* was achieved using the Ribo^TM^ Smart Silencer designed and synthesized in Ribobio (China). It contained three siRNA and three antisense oligonucleotides (ASO) targeting separate sequences. Lipofectamine RNAiMAX (Invitrogen, USA) was utilized as the transfection reagent to deliver the smart silencer following the protocol. To be specific, 2 × 10^5 cells were seeded in the 24-well plates and Smart Silencer of *NEAT1* (200 nM) together with RNAiMAX (3 μL) were used for transfection. They were diluted in the 50 μL serum-free medium separately. After being mixed at a 1:1 ratio and incubated for five minutes, they were added into the plates. Adenovirus vectors carrying the sequences of *NEAT1*, *miR-365a-3p* mimics, and *miR-365a-3p* inhibitor were constructed via the AdEasy system (Hanbio, China) and the multiplicity of infection (MOI) was 300. MoDCs transfected with empty vectors were considered as controls. Transfected cells were incubated for another 48 hours before they were collected for further analysis.

### ELISA

Using a commercially available Human ELISA IL-6 DuoSet from R&D (USA), the protein levels of IL-6 in plasma and cell culture supernatant were measured following the manufacturer’s instructions. MoDCs were stimulated with LPS (1ug/mL) for 24 hours for maturation and the supernatants were collected for ELISA.

### Flow-cytometry assay

Fluorochrome-labelled antibodies for CD14 (PE), CD40 (Alexa Fluor 700), CD83 (PE/Dazzle), CD11c (APC), and CD86 (Brilliant Violet 510) were purchased from Biolegend (USA). Antibodies for HLA-DR (PE-Cy7) were purchased from Invitrogen (USA). Collected moDCs were first incubated with viability dye FVD780 (eBioscience, USA) for 15 minutes on ice and washed twice with DPBS. Fc blocker (Biolegend, USA) was added for another 15 minutes of incubation to exclude unspecified antigen-binding. Then, the mixture of targeted antibodies was added and incubated for 30 minutes. All samples were processed with the Fortessa flow cytometer (BD, USA), and data were analysed with FlowJo. Unstained controls were used to set up instruments and determine voltages, single stained samples were also prepared for compensation controls and to reveal the fluorescence spillover. In addition, fluorescence minus one (FMO) controls were included in the protocol to help define positive from negative populations.

### Statistical analysis

Statistical comparisons were performed using Student’s *t*-test, one-way ANOVA, nonparametric Mann–Whitney test, or nonparametric Kruskal–Wallis test. Parametric correlation (Pearson) was utilized to determine underlying associations between *NEAT1* expression and SLEDAI score. Three separate independent repeats were made for each experiment. Data were analysed with GraphPad Prism and presented as mean ± standard error of the mean (SEM). Statistical significance was defined as a P-value below 0.05.

## Results

### The levels of *NEAT1* and IL-6 are elevated in SLE patients compared with healthy controls

**Figure 1.**
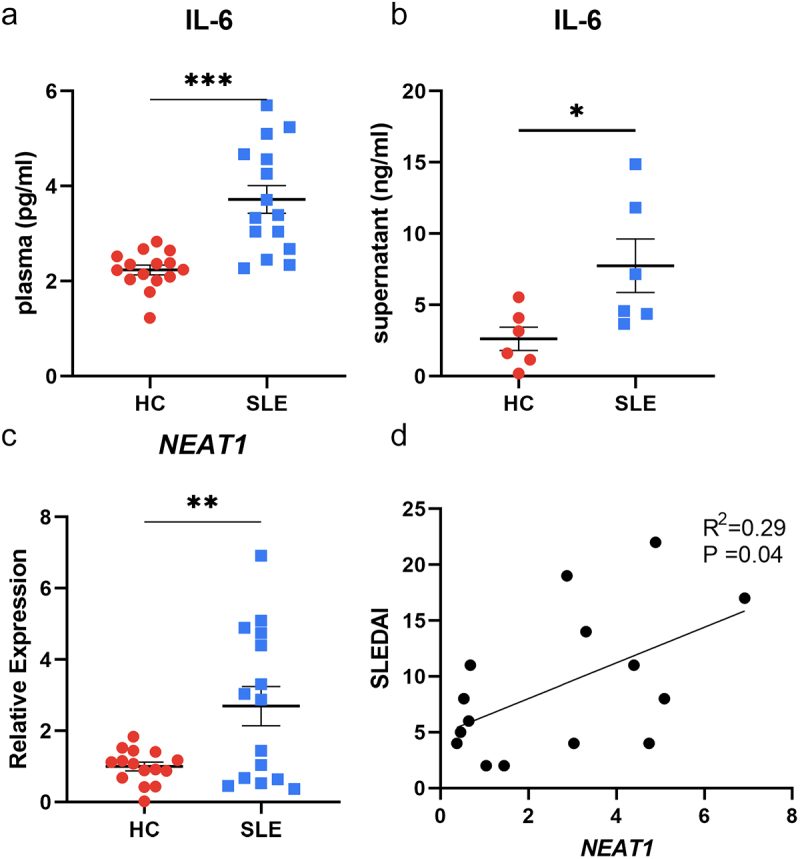
The levels of *NEAT1* and IL-6 are elevated in SLE patients compared with HCs. (A) The comparison of secreted levels of IL-6 in plasma between SLE patients and HCs (15 vs 15). (B) The comparison of secreted levels of IL-6 in the moDCs cell supernatants between SLE patients and HCs (6 vs 6). (C) The comparison of the expression level of *NEAT1* between SLE patients and HCs (15 vs 15). (D) The correlation between the expression level of *NEAT1* and SLEDAI scores of SLE patients. HC, healthy controls; moDCs, monocyte-derived dendritic cells; SLE: systemic lupus erythematosus; *NEAT1*, nuclear paraspeckle assembly transcript 1; SLEDAI: systemic lupus erythematosus disease activity index. *P* values for two parametric sample comparisons were determined by unpaired t-test and the Pearson correlation coefficient was calculated.

Monocytes were acquired from both SLE patients and healthy controls, and they were cultivated towards moDCs in vitro. Detailed clinical and laboratory characteristics are summarized in Table 1 and S2. Surface markers including CD14, CD11c, CD40, CD83, CD86, and HLA-DR of matured moDCs were analysed by flow cytometry (Figure S1), and the expression pattern was consistent with the published studies [20,21]. The secreted IL-6 was measured in the plasma samples of all subjects enrolled, and significantly higher levels of IL-6 were found among patients with SLE compared to healthy individuals (3.72 ± 0.29 pg/mL vs 2.24 ± 0.10 pg/mL, *P* < 0.001; Figure 1A). The trend was similar when IL-6 secretions in moDCs supernatants were compared between SLE patients and healthy controls (7.75 ± 1.88 ng/mL vs 2.63 ± 0.82 ng/mL, *P* < 0.05; Figure 1B). In addition, the *NEAT1* expression levels were significantly elevated in moDCs of SLE patients (*P* < 0.01; Figure 1C). Furthermore, there was a positive correlation between *NEAT1* expressions and SLEDAI scores in SLE participants (R^2^ = 0.29, *P* = 0.04; Figure 1D).

*p <0.05, **p <0.01, ***p <0.001.

**Table 1.** Clinical characteristics of included SLE patients.

Characteristics	SLE(*n* = 15)	HC(*n* = 15)
Sex, male/female(n)	0/15	0/15
Age (years)	41.6 ± 3.05	37.2 ± 3.29
Duration (months)	3(1–192)	
RBC (10^12)	3.79 ± 0.18	
WBC (10^9)	4.11 (2.42–10.54)	
Hemoglobin (g/L)	111.8 ± 5.31	
Platelet count (10^9)	184.8 ± 16.62	
ESR (mm/h)	23 ± 4.4	
Urine protein, yes/no(n)	4/11	
SLEDAI score	9.13 ± 1.64	
ANA >1:320, yes/no(n)	15/0	
Anti-dsDNA (IU/ml)	393.9 ± 75.16	
Abnormal (low) complement C3, yes/no(n)	8/7	
Abnormal (low) complement C4, yes/no(n)	8/7	
Organ involvement, yes/no(n)	11/4	
Glucocorticoids^a^, yes/no(n)	13/2	
Immunosuppressive drugs, yes/no(n)	1/14	

Data are presented as median (minimum-maximum) or mean ± SEM. ^a^:systemic usage of glucocorticoids within one year of enrolment. ANA: antinuclear antibody; SLE: systemic lupus erythematosus; SLEDAI: systemic lupus erythematosus disease activity index; RBC: red blood cell; WBC: white blood cell; ESR: erythrocyte sedimentation rate; HC: healthy controls.

### *NEAT1* is closely associated with inflammatory functions of moDcs.

**Figure 2.**
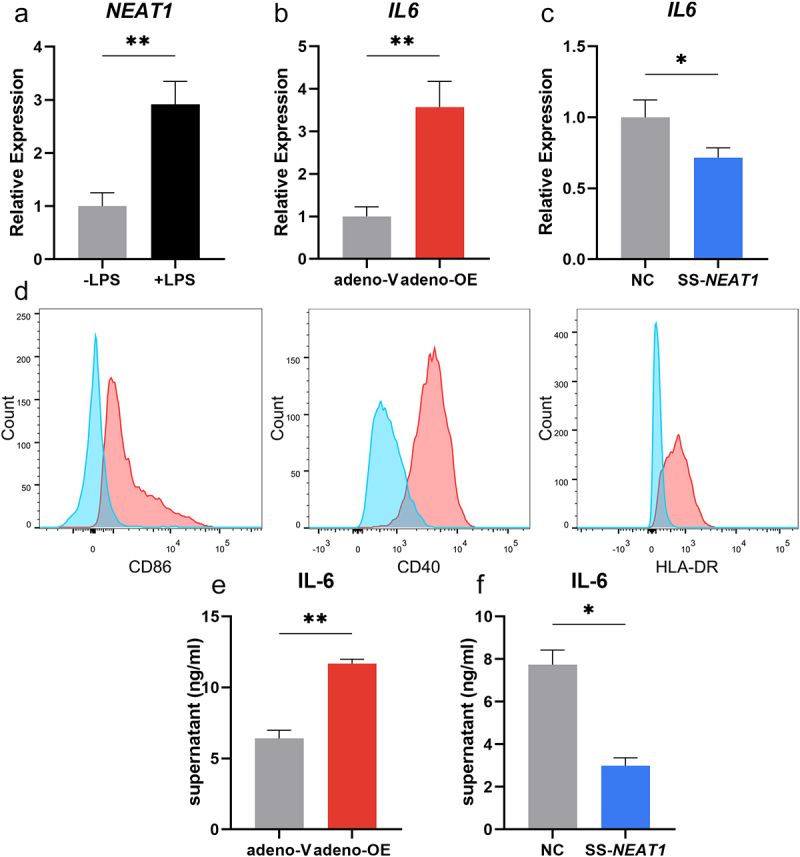
*NEAT1* is closely associated with the inflammatory functions of moDcs. (A) The change in the expression level of *NEAT1* when the stimulator LPS was added for 24 hours. (B-C) Expression levels of mRNA *IL6* in moDCs when *NEAT1* was overexpressed (adeno-V vs adeno-OE) and knocked down (NC vs SS-*NEAT1*). (D) Expressions of surface markers including CD86, CD40, and HLA-DR in the *NEAT1* overexpression group (red) compared with negative controls (blue). (E-F) The secretion of IL-6 in the supernatant of moDCs when *NEAT1* was up-regulated (adeno-V vs adeno-OE) and downregulated (NC vs SS-*NEAT1*). Data were shown as mean±SEM. moDCs, monocyte-derived dendritic cells; NC, negative control; *NEAT1*, nuclear paraspeckle assembly transcript 1; SS, smart silencer; adeno, adenovirus; OE, overexpression. *P* values for two parametric sample comparisons were determined by unpaired t-test.

It was observed that the expression level of *NEAT1* in moDCs increased significantly when LPS was added as the stimulator for 24 hours (*P* < 0.01; Figure 2A). Adenovirus containing the sequence of *NEAT1* was transfected into moDCs, and up-regulation of *NEAT1* was then confirmed by qRT-PCR (*P* < 0.05; Figure S2A). mRNA expression of *IL6* increased along with the expression of *NEAT1* (*P* < 0.01; Figure 2B). Knock-down of *NEAT1* was accomplished by the smart silencer consisting of siRNAs and ASOs and was followed by decreased *IL6* expression levels (*P* < 0.05; Figure S2B and 2C). Similarly, co-stimulatory surface makers on matured moDCs including CD86, CD40, and HLA-DR in the *NEAT1* overexpression group became upregulated compared to controls (Figure 2D). The comparisons of secreted IL-6 in the supernatants from moDCs were made between adeno-V, adeno-OE, SS-NC, and SS-*NEAT1* groups by ELISA (Figure 2E-F). Other inflammation-related cytokine expressions including *IL1B, TNFA, TGFB, IL10*, and *IL12A* were also measured, but none of them exhibited consistently significant changes between separate *NEAT1* expression level groups (Figure S3).

*p < 0.05, **p < 0.01.

### *MiR-365a-3p* could reduce IL-6 secretion in moDcs

**Figure 3.**
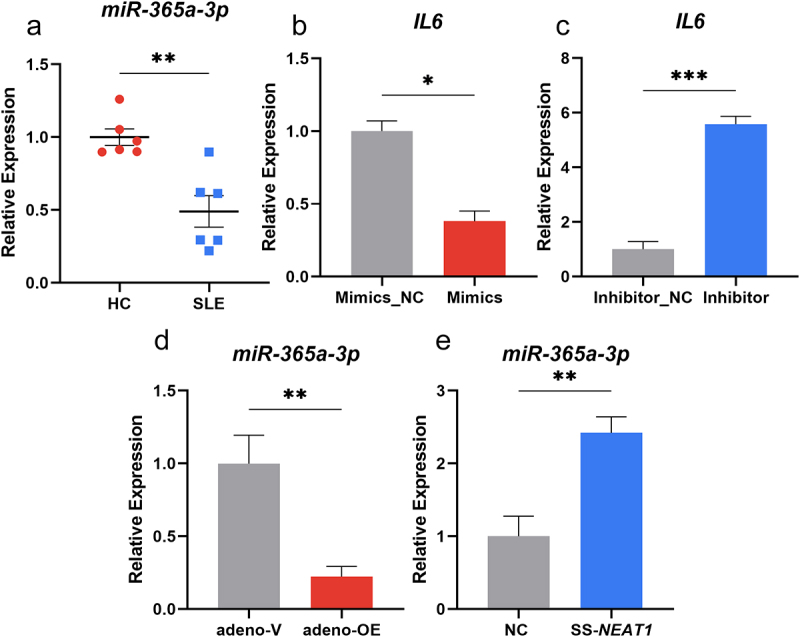
Mir-365a-3p could reduce IL-6 secretion in moDcs. (A) Expression levels of *miR-365a-3p* in moDCs of SLE patients compared with those of HCs (6 vs 6). (B-C) Adenovirus containing *miR-365a-3p* mimics and inhibitor were transfected into moDCs and the mRNA levels of *IL6* changed as *miR-365a-3p* was artificially modulated. (D-E) The levels of *miR-365a-3p* were validated by qRT-PCR when *NEAT1* was knocked down and up-regulated. moDCs, monocyte-derived dendritic cells; NC, negative control; *NEAT1*, nuclear paraspeckle assembly transcript 1; SS, smart silencer; adeno, adenovirus. *P* values for two sample comparisons were determined by unpaired t-test or Mann–Whitney test.

In order to further analyse how *NEAT1* influences the secretion of IL-6 in moDCs, bioinformatic analysis were performed and the ENCORI platform (https://starbase.sysu.edu.cn/) was utilized. *MiR-365a-3p* was identified to contain the sequence sites which can bind with the 3’ UTR region of *IL6* as well as *NEAT1*. A significant decrease was also shown in the expression levels of *miR-365a-3p* in moDCs of patients compared to controls (*P* < 0.01; Figure 3A). To verify its influence on the secretion of IL-6 in moDCs, microRNA mimics, and inhibitor were packed into the adenoviruses for transfection and then the expression level of *miR-365a-3p* was changed (Figure S2C-D). It was observed that when the level of *miR-365a-3p* was over-regulated, the mRNA expression of *IL6* decreased significantly (*P* < 0.05), while the *miR-365a-3p* inhibitor transfection resulted in increased expression of *IL6* (*P* < 0.001) (Figure 3B-C). Thus, *miR-365a-3p* could act as a negative effector on *IL6* expression. The effect of *NEAT1* on *miR-365a-3p* expression in moDCs was investigated using qRT-PCR, and overexpression of *NEAT1* resulted in a decrease in *miR-365a-3p* expression (*P* < 0.01) (Figure 3D). Furthermore, *miR-365a-3p* expression was considerably elevated in moDCs when *NEAT1* was downregulated (*P* < 0.01) (Figure 3E).

*p < 0.05, **p < 0.01, ***p < 0.001.

### *NEAT1* can act as the competing endogenous RNA for *miR-365a-3p* and *IL6*

**Figure 4.**
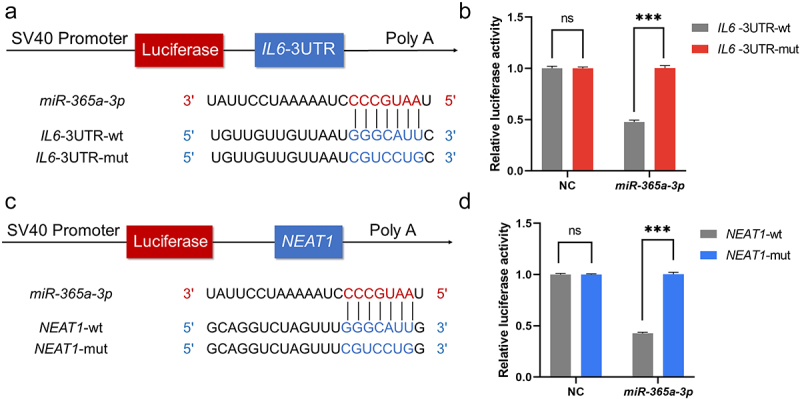
*NEAT1* can act as the competing endogenous RNA for *miR-365a-3p* and *IL6*. (A, C) pSI-Check2 plasmids carrying predicted binding sites of *NEAT1* and *IL6* with *miR-365a-3p* and corresponding mutants were constructed. Dual luciferase reporter assays were performed. (B, D) *MiR-365a-3p* mimics and negative controls were added separately to interact with wildtype and mutant sequences of *NEAT1* and 3’UTR of *IL6*. NC, negative control; *NEAT1*, nuclear paraspeckle assembly transcript 1; wt, wildtype; mut, mutant; miR, microRNA, ns, not significant. *P* values for two parametric sample comparisons were determined by unpaired t-test.

Based on the underlying binding sites predicted by the ENCORI platform, plasmids containing wildtype and mutant sequences of *NEAT1* and 3’UTR of *IL6* were transfected, and dual-luciferase reporter assays were used to verify if *miR-365a-3p* can bind to the expected target locations (Figure 4A-C). Results showed that *miR-365a-3p* significantly reduced the luciferase activity of cells transfected with *IL6*-wt rather than *IL6*-mut, indicating a direct interaction between *miR-365a-3p* and the 3‘UTR of *IL6* (Figure 4B). The predicted binding sites between *NEAT1* and *miR-365a-3p* were likewise validated by the assay. Luciferase expression levels of the *miR-365a-3p* mimic and *NEAT1*-wt plasmid co-transfection group were significantly lower than those of the *NEAT1*-mut group after 48 hours, while for the NC mimic, luciferase activity was unaffected (Figure 4D).

***p <0.001.

### *NEAT1* restores the IL-6 secretion by targeting *miR-365a-3p*

**Figure 5.**
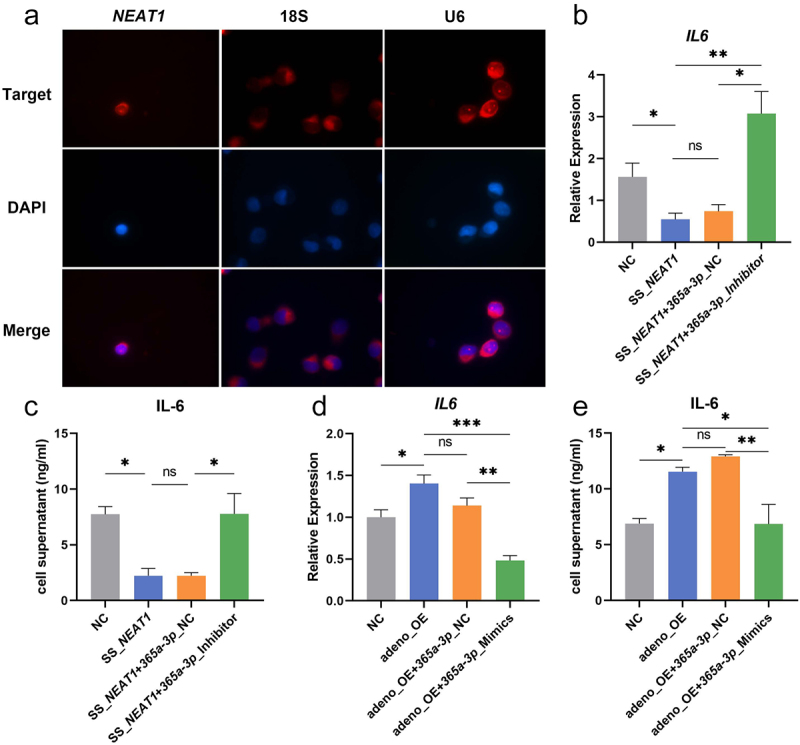
*NEAT1* restores the IL-6 secretion by targeting *miR-365a-3p*. (A) Intracellular colocalization of *NEAT1* in moDCs was ascertained using RNA FISH. 18s and U6 were the controls for the cytoplasm and nucleus. (B-E) The secretion and mRNA levels of IL-6 were further evaluated by ELISA and qRT-PCR in rescue validations when *NEAT1* and *miR-365a-3p* were regulated at the same time. Data were shown as mean±SEM. FISH, Fluorescence in situ hybridization; NC, negative control; *NEAT1*, nuclear paraspeckle assembly transcript 1; SS, smart silencer; adeno, adenovirus; OE, overexpression; ns, not significant. The parametric one-way ANOVA test or nonparametric Kruskal–Wallis test were applied for the multiple sample comparisons.

Sub-localization of *NEAT1* in moDCs was investigated using the FISH probe and it was found that *NEAT1* was mainly located in the nucleus (Figure 5A). Rescue experiments were then carried out where the down regulation of IL-6 by knock down of *NEAT1* could be altered when *miR-365a-3p* inhibitor was transfected into moDCs (Figure 5B-C). Similarly, the adeno-OE + *miR-365a-3p* mimics group secreted less IL-6 compared with the adeno-OE group (Figure 5D-E). The restored influence of *NEAT1* was confirmed by both ELISA and qRT-PCR.

*p < 0.05, **p < 0.01, ***p < 0.001.

## Discussion

SLE is a potentially fatal autoimmune disease with a remission and flare pattern. Lupus patients may complain of heterogeneous symptoms, from minor manifestations to life-threatening kidney or brain involvement [22]. In the past 30 years, therapeutical advancements have significantly improved the life expectancy and quality of life of lupus patients. However, there are still some major issues in the treatment of lupus patients, particularly for SLE [23]. These include adverse effects, such as osteoporosis, gastrointestinal side effects, infection, and cardiovascular disease, associated with corticosteroids and immunosuppressive treatments [24]. Moreover, even after receiving these conventional treatments, a certain percentage of patients fail to respond. Thus, there have been a number of emerging biological therapies for SLE patients during the last decade [25]. These therapies can be briefly categorized into B-cell target therapies, T-cell target therapies, and anti-cytokines therapies such as IL-6, TNF-α, and IFN-α [26].

IL-6 is an inflammation marker in certain autoimmune diseases, which plays a critical role in the recruitment of inflammatory cells and the modulation of the immune system [27]. In SLE, IL-6 levels in the serum or plasma of patients are much higher than those of healthy controls, according to a number of studies, and are associated with disease activity and other critical standard biomarkers [28,29]. A previous study found that IL-6 generated by DCs was essential for B cell differentiation into plasma cells and antibody production [30]. Furthermore, in SLE murine model, IL-6 deficient mice had a delayed onset of lupus nephritis, enhanced kidney function, and a longer lifespan [31]. These results suggest that IL-6 produced by DCs may potentially influence the initiation and progression of SLE.

In our study, it was observed that IL-6 increased in both the SLE plasma and cell supernatants of moDCs, which is consistent with other research. For example, a meta-analysis concluded that *IL6* promoter variants can be a risk factor and have correlations with SLE disease progression and pathogenesis [32]. IgG anti-DNA and chromatin antibodies in mice presenting with lupus symptoms are strictly IL-6 dependent, indicating the roles of IL-6 in antibody production [33]. Based on the existing knowledge of the role of IL-6 in autoimmune illnesses, biological antibodies targeting IL-6 have been developed with the goal of alleviating disease by lowering the level of this pro-inflammatory cytokine [34]. Tocilizumab, a humanized anti-IL-6 R monoclonal antibody, for example, was the first drug to successfully block the IL-6 signal and was licenced by the US Food and Drug Administration (FDA) for the treatment of rheumatoid arthritis, systemic juvenile idiopathic and giant cell arteritis [35,36]. Tocilizumab reduced illness severity in patients diagnosed as moderately active SLE in an open-label phase I study, however, neutropenia and infection emerged in a dose-dependent manner [37]. Nonetheless, tocilizumab has been shown to be effective in treating SLE flares in adult patients in multiple case reports [38,39].

In addition, our study has also revealed that lncRNA *NEAT1* could promote the secretion of IL-6 in moDCs while *miR-365a-3p* mainly functions as a negative modulator. These two makers could serve as new targets to modulate IL-6 generation or DC’s biological functions, thus refreshing the significance of IL-6 and DCs in SLE treatment.

LncRNAs can regulate gene expression by acting as competing endogenous RNAs [40]. According to this hypothesis, some lncRNAs can impair microRNAs’ activity by sequestering microRNAs, hence upregulating miRNA target gene expression [41]. A group of lncRNAs, such as HOTAIR, H19, and HULC, have been identified by both bioinformatic prediction and experimental verification to function as ceRNAs, mainly in carcinogenesis [42–44]. Our study has found that lncRNA *NEAT1* could efficiently interact with *miR-365a–3p* to modulate the expression of *IL6* in moDCs, and their binding sequences were confirmed by dual-luciferase-reporter gene assay. *NEAT1* is a crucial structural element of the paraspeckle nuclear domain, which regulates genes predominantly through the nuclear retention of proteins and RNAs, according to earlier studies [45]. Many studies have identified *NEAT1’*s aberrant expression and prognostic relevance in a variety of tumors; the majority of them define *NEAT1* as an oncogene that is overexpressed in tumors compared with normal tissues and it commonly boosts tumor cell growth [18]. In autoimmune diseases, a recent study has revealed that in rheumatoid arthritis, *NEAT1* enhances cell proliferation, migration, invasion, and the release of inflammatory cytokines, produces higher S-to-G2/M phase transition, and inhibits apoptosis in fibroblast-like synoviocytes [46]. Furthermore, *NEAT1* can also switch its subcellular position from the nucleus to the cytoplasm to influence inflammasome activation in macrophages, and it plays an important role in innate immunity by regulating caspase-1 assembly and stability [19]. Zhang et al. have previously confirmed that the expression of *NEAT1* was positively correlated with SLE activity and it could activate TLR4-mediated inflammation via the MAPK signaling pathway in monocytes [47]. Our study further validated *NEAT1*s role in dendritic cells which are of critical importance in autoimmunity, and we discovered that *NEAT1* could function as the ceRNA to modulate IL-6 secretion via microRNA. We believe that these findings contribute to a better understanding of the role of *NEAT1* in immune cell biology and give insight into its potential as a promising target for future research in SLE.

Although the present study has confirmed that *NEAT1* and IL-6 are differently expressed in SLE and that *NEAT1* targets IL-6, it should be noted that potential additional clinical correlations between *NEAT1* and SLE need to be investigated, as well as the relationship of SLEDAI scores with *NEAT1* and IL-6 secretion. Due to underlying differences in the gene expression and cytokine secretion profiles of moDCs from SLE patients, more blood samples from patients need to be included to fully ascertain the roles of *NEAT1* in moDCs. Nevertheless, the interactions between *NEAT1* and IL-6 could be only one part of the complex pathogenesis in SLE, and studies carried out solely on moDCs from healthy controls could help to get rid of other underlying interferences and focus on the illustration of the correlation between *NEAT1* and IL-6.

## Supplementary Material

Supplemental Material

## Data Availability

The authors confirm that the data supporting the findings of this study are available within the article and its supplementary materials.

## References

[1] Tian J, Zhang D, Yao X, et al. Global epidemiology of systemic lupus erythematosus: a comprehensive systematic analysis and modelling study. Ann Rheum Dis. 2023;82(3):351–12. doi:10.1136/ard-2022-223035 36241363 PMC9933169

[2] Rees F, Doherty M, Grainge MJ, et al. The worldwide incidence and prevalence of systemic lupus erythematosus: a systematic review of epidemiological studies. Rheumatology. 2017;56(11):1945–1961. doi:10.1093/rheumatology/kex260 28968809

[3] Melander C, Sallée M, Trolliet P, et al. Rituximab in severe lupus nephritis: early b-cell depletion affects long-term renal outcome. Clin J Am Soc Nephrol. 2009;4(3):579–587. doi:10.2215/CJN.04030808 19261822 PMC2653670

[4] Liossis S-N, Staveri C. B cell-based treatments in SLE: past experience and current directions. Curr Rheumatol Rep. 2017;19(12):78. doi: 10.1007/s11926-017-0707-z 29101479

[5] Herrada AA, Escobedo N, Iruretagoyena M, et al. Innate immune cells’ contribution to systemic lupus erythematosus. Front Immunol. 2019;10:772. doi: 10.3389/fimmu.2019.00772 31037070 PMC6476281

[6] Blanco P, Palucka A, Pascual V, et al. Dendritic cells and cytokines in human inflammatory and autoimmune diseases. Cytokine Growth Factor Rev. 2008;19(1):41–52. doi:10.1016/j.cytogfr.2007.10.004 18258476 PMC2413068

[7] Eriksson U, Ricci R, Hunziker L, et al. Dendritic cell–induced autoimmune heart failure requires cooperation between adaptive and innate immunity. Nat Med. 2003;9(12):1484–1490. doi:10.1038/nm960 14625544

[8] Bell GM, Anderson AE, Diboll J, et al. Autologous tolerogenic dendritic cells for rheumatoid and inflammatory arthritis. Ann Rheum Dis. 2017;76(1):227–234. doi:10.1136/annrheumdis-2015-208456 27117700 PMC5264217

[9] Phillips BE, Garciafigueroa Y, Engman C, et al. Tolerogenic dendritic cells and T-Regulatory cells at the clinical trials crossroad for the treatment of autoimmune disease; emphasis on type 1 diabetes therapy. Front Immunol. 2019;10:148. doi: 10.3389/fimmu.2019.00148 30787930 PMC6372505

[10] Ding D, Mehta H, McCune WJ, et al. Aberrant phenotype and function of myeloid dendritic cells in systemic lupus erythematosus. J Immunol. 2006;177(9):5878–5889. doi:10.4049/jimmunol.177.9.5878 17056512

[11] Wang Y, Liang J, Qin H, et al. Elevated expression of miR-142-3p is related to the pro-inflammatory function of monocyte-derived dendritic cells in SLE. Arthritis Res Ther. 2016;18(1):263. doi:10.1186/s13075-016-1158-z 27852285 PMC5112667

[12] Rinn JL, Chang HY. Genome regulation by long noncoding RNAs. Annu Rev Biochem. 2012;81(1):145–166. doi: 10.1146/annurev-biochem-051410-092902 22663078 PMC3858397

[13] Niu L, Lou F, Sun Y, et al. A micropeptide encoded by lncRNA MIR155HG suppresses autoimmune inflammation via modulating antigen presentation. Sci Adv. 2020;6(21):eaaz2059. doi:10.1126/sciadv.aaz2059 32671205 PMC7314557

[14] Wang P, Xue Y, Han Y, et al. The STAT3-binding long noncoding RNA lnc-DC controls human dendritic cell differentiation. Science. 2014;344(6181):310–313. doi:10.1126/science.1251456 24744378

[15] Liu J, Zhang X, Chen K, et al. CCR7 chemokine receptor-inducible lnc-Dpf3 restrains dendritic cell migration by inhibiting HIF-1α-mediated glycolysis. Immunity. 2019;50(3):600–615.e15. doi:10.1016/j.immuni.2019.01.021 30824325

[16] Salmena L, Poliseno L, Tay Y, et al. A ceRNA hypothesis: the rosetta stone of a hidden RNA language? Cell. 2011;146(3):353–358. doi:10.1016/j.cell.2011.07.014 21802130 PMC3235919

[17] Wang Y, Chen S, Chen S, et al. Long noncoding RNA expression profile and association with SLEDAI score in monocyte-derived dendritic cells from patients with systematic lupus erythematosus. Arthritis Res Ther. 2018;20(1):138. doi:10.1186/s13075-018-1640-x 29996948 PMC6042324

[18] Li S, Li J, Chen C, et al. Pan-cancer analysis of long non-coding RNA NEAT1 in various cancers. Genes Dis. 2018;5(1):27–35. doi:10.1016/j.gendis.2017.11.003 30258932 PMC6146416

[19] Zhang P, Cao L, Zhou R, et al. The lncRNA Neat1 promotes activation of inflammasomes in macrophages. Nat Commun. 2019;10(1):1495. doi:10.1038/s41467-019-09482-6 30940803 PMC6445148

[20] Waeckerle-Men Y, Scandella E, Allmen E, et al. Phenotype and functional analysis of human monocyte-derived dendritic cells loaded with biodegradable poly(lactide-co-glycolide) microspheres for immunotherapy. J Immunol Methods. 2004;287(1–2):109–124. doi:10.1016/j.jim.2004.01.010 15099760

[21] Martin H, Laborel-Préneron E, Fraysse F, et al. Aquaphilus dolomiae extract counteracts the effects of cutaneous S. aureus secretome isolated from atopic children on CD4 + T cell activation. Pharm Biol. 2016;54(11):2782–2785. doi:10.3109/13880209.2016.1173069 27180655

[22] Shaban A, Leira EC. Neurological complications in patients with systemic lupus erythematosus. Curr Neurol Neurosci Rep. 2019;19(12):97. doi: 10.1007/s11910-019-1012-1 31773306

[23] Mohamed A, Chen Y, Wu H, et al. Therapeutic advances in the treatment of SLE. Int Immunopharmacol. 2019;72:218–223. doi: 10.1016/j.intimp.2019.03.010 31002998

[24] Oon S, Wilson NJ, Wicks I. Targeted therapeutics in SLE: emerging strategies to modulate the interferon pathway. Clin Transl Immunol. 2016;5(5):e79. doi: 10.1038/cti.2016.26 PMC4910120 27350879

[25] Mathias LM, Stohl W. Systemic lupus erythematosus (SLE): emerging therapeutic targets. Expert Opin Ther Targets. 2020;24(12):1283–1302. doi: 10.1080/14728222.2020.1832464 33034541

[26] Rönnblom L, Elkon KB. Cytokines as therapeutic targets in SLE. Nat Rev Rheumatol. 2010;6(6):339–347. doi: 10.1038/nrrheum.2010.64 20440285

[27] Yao X, Huang J, Zhong H, et al. Targeting interleukin-6 in inflammatory autoimmune diseases and cancers. Pharmacol Ther. 2014;141(2):125–139. doi:10.1016/j.pharmthera.2013.09.004 24076269

[28] Jin S, Yu C, Yu B. Changes of serum IL-6, IL-10 and TNF-α levels in patients with systemic lupus erythematosus and their clinical value. Am J Transl Res. 2021;13(4):2867–2874. 34017450 PMC8129414

[29] Talaat RM, Mohamed SF, Bassyouni IH, et al. Th1/Th2/Th17/Treg cytokine imbalance in systemic lupus erythematosus (SLE) patients: correlation with disease activity. Cytokine. 2015;72(2):146–153. doi:10.1016/j.cyto.2014.12.027 25647269

[30] Jego G, Palucka AK, Blanck J-P, et al. Plasmacytoid dendritic cells induce plasma cell differentiation through Type I interferon and interleukin 6. Immunity. 2003;19(2):225–234. doi:10.1016/S1074-7613(03)00208-5 12932356

[31] Cash H, Relle M, Menke J, et al. Interleukin 6 (IL-6) deficiency delays lupus nephritis in MRL- *Fas ^lpr^* mice: the iL-6 pathway as a new therapeutic target in treatment of autoimmune kidney disease in systemic lupus erythematosus. J Rheumatol. 2010;37(1):60–70. doi:10.3899/jrheum.090194 19955044

[32] Katkam SK, Rajasekhar L, Kumaraswami K, et al. Association of IL - 6 -174 G>C polymorphism with the risk of SLE among south Indians: evidence from case–control study and meta-analysis. Lupus. 2017;26:1491–1501. doi: 10.1177/0961203317711010 28530463

[33] Richards HB, Satoh M, Shaw M, et al. Interleukin 6 dependence of Anti-DNA antibody production: evidence for two pathways of autoantibody formation in pristane-induced lupus. J Exp Med. 1998;188(5):985–990. doi:10.1084/jem.188.5.985 9730900 PMC2213386

[34] Jung J-Y, Kim M-Y, Suh C-H, et al. Off-label use of tocilizumab to treat non-juvenile idiopathic arthritis in pediatric rheumatic patients: a literature review. Pediatr Rheumatol. 2018;16(1):79. doi:10.1186/s12969-018-0296-z PMC6295005 30547812

[35] Scott LJ. Tocilizumab: a review in rheumatoid arthritis. Drugs. 2017;77(17):1865–1879. doi: 10.1007/s40265-017-0829-7 29094311 PMC5736769

[36] Stone JH, Tuckwell K, Dimonaco S, et al. Trial of tocilizumab in giant-cell arteritis. N Engl J Med. 2017;377(4):317–328. doi:10.1056/NEJMoa1613849 28745999

[37] Illei GG, Shirota Y, Yarboro CH, et al. Tocilizumab in systemic lupus erythematosus: data on safety, preliminary efficacy, and impact on circulating plasma cells from an open-label phase I dosage-escalation study. Arthritis Rheum. 2010;62(2):542–552. doi:10.1002/art.27221 20112381 PMC3057537

[38] Hirooka Y, Okuda S, Sugiyama M, et al. Case report: a rare case of elderly-onset adult-onset still’s disease in a patient with systemic lupus erythematosus. Front Immunol. 2022;13:822169. doi: 10.3389/fimmu.2022.822169 35116046 PMC8803898

[39] De Matteis A, Sacco E, Celani C, et al. Tocilizumab for massive refractory pleural effusion in an adolescent with systemic lupus erythematosus. Pediatr Rheumatol. 2021;19(1):144. doi:10.1186/s12969-021-00635-w PMC8444491 34530845

[40] Thomson DW, Dinger ME. Endogenous microRNA sponges: evidence and controversy. Nat Rev Genet. 2016;17(5):272–283. doi: 10.1038/nrg.2016.20 27040487

[41] Yamamura S, Imai-Sumida M, Tanaka Y, et al. Interaction and cross-talk between non-coding RNAs. Cell Mol Life Sci. 2018;75(3):467–484. doi:10.1007/s00018-017-2626-6 28840253 PMC5765200

[42] Liu X, Sun M, Nie F, et al. Lnc RNA HOTAIR functions as a competing endogenous RNA to regulate HER2 expression by sponging miR-331-3p in gastric cancer. Mol Cancer. 2014;13(1):92. doi:10.1186/1476-4598-13-92 24775712 PMC4021402

[43] Imig J, Brunschweiger A, Brümmer A, et al. MiR-CLIP capture of a miRNA targetome uncovers a lincRNA H19–miR-106a interaction. Nat Chem Biol. 2015;11(2):107–114. doi:10.1038/nchembio.1713 25531890

[44] Wang J, Liu X, Wu H, et al. CREB up-regulates long non-coding RNA, HULC expression through interaction with microRNA-372 in liver cancer. Nucleic Acids Res. 2010;38(16):5366–5383. doi:10.1093/nar/gkq285 20423907 PMC2938198

[45] Bond CS, Fox AH. Paraspeckles: nuclear bodies built on long noncoding RNA. J Cell Bio. 2009;186(5):637–644. doi: 10.1083/jcb.200906113 19720872 PMC2742191

[46] Wang Y, Hou L, Yuan X, et al. LncRNA NEAT1 targets fibroblast-like synoviocytes in rheumatoid arthritis via the miR-410-3p/YY1 axis. Front Immunol. 2020;11:1975. doi: 10.3389/fimmu.2020.01975 32983133 PMC7485383

[47] Zhang F, Wu L, Qian J, et al. Identification of the long noncoding RNA NEAT1 as a novel inflammatory regulator acting through MAPK pathway in human lupus. J Autoimmun. 2016;75:96–104. doi: 10.1016/j.jaut.2016.07.012 27481557

