# Supplementary material for: LncRNA NEAT1 promotes IL-6 secretion in monocyte-derived dendritic cells via sponging miR-365a-3p in systemic lupus erythematosus
Source: Epigenetics. 2023 Jun 21;18(1):2226492. doi: 10.1080/15592294.2023.2226492 (PMC10286691; doi:10.1080/15592294.2023.2226492)
Supplement: Supplemental Material [file KEPI_A_2226492_SM3856.docx]

**Figure S1.** **Validation characteristic of monocyte-derived dendritic cells.** (A-F) Surface markers on monocyte-derived dendritic cells (moDCs) were measured by flow cytometry. (G) Morphology of mature moDCs after being cultured for 8 days in vitro (magnification: 200fold).


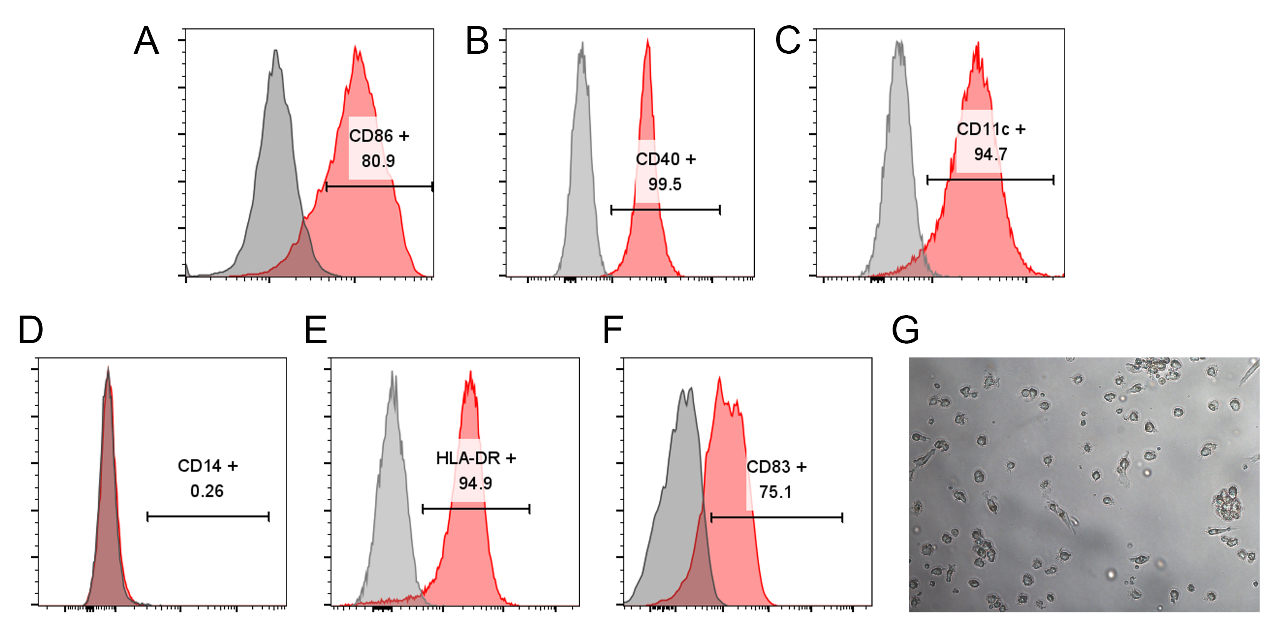


**Figure S2.** **Transfection efficacy verification of *NEAT1* and *miR-365a-3p*.** (A-B) *NEAT1* levels were artificially regulated after transfection of adenovirus and smart silencer. (C-D) The expression levels of *miR-365a-3p* when micro mimics and inhibitor were transferred. NC, negative control; *NEAT1*, nuclear paraspeckle assembly transcript 1; SS, smart silencer; adeno, adenovirus; OE, overexpression. P values for two sample comparisons were determined by unpaired t-test. ^*^p<0.05, ^**^p<0.01, ^***^p<0.001,^****^p<0.0001.

**
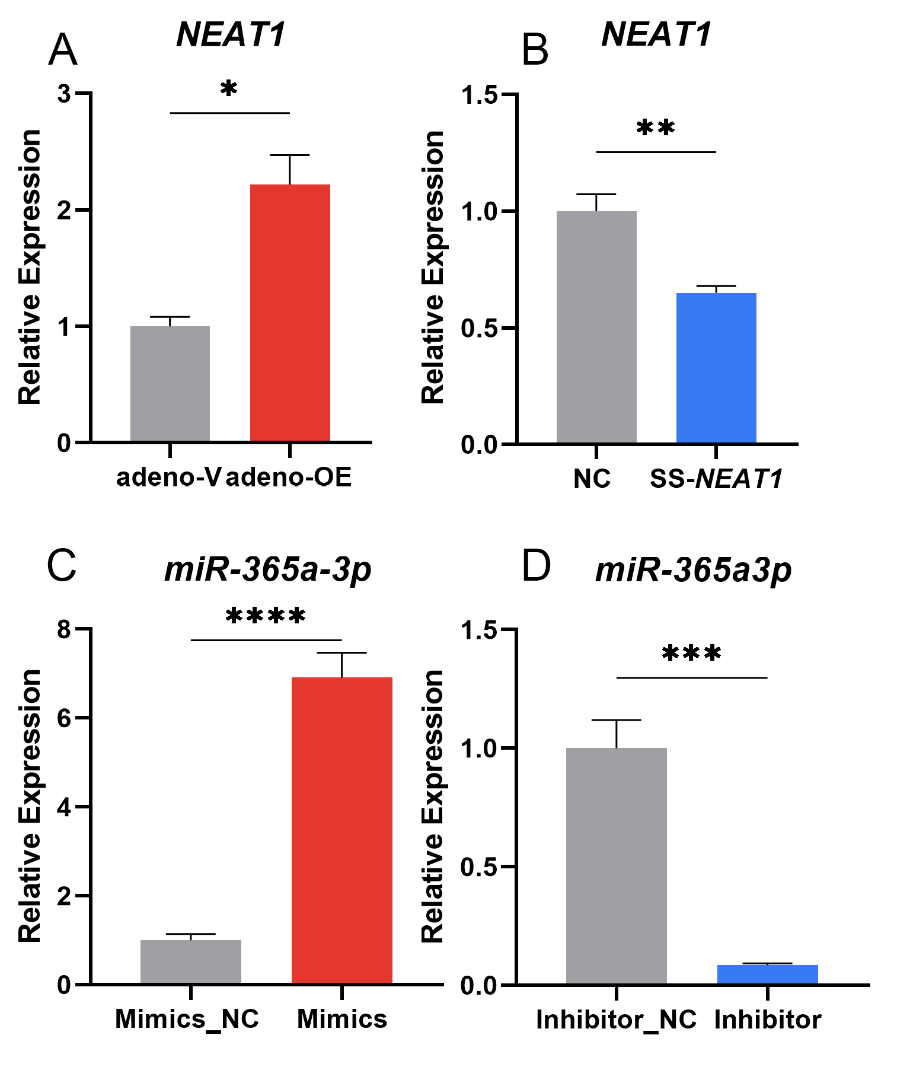
**

**Figure S3.** **Expression levels of inflammation-related cytokines when *NEAT1* levels were altered.** *IL10, IL12A, IL1B, TGFB,* and *TNFA* were compared between adeno-V vs adeno-OE (A) and NC vs SS-*NEAT1*(B) by qRT-PCR. Data were shown as mean ± SEM. NC, negative control; *NEAT1*, nuclear paraspeckle assembly transcript 1; SS, smart silencer; adeno, adenovirus; OE, overexpression. P values for two sample comparisons were determined by unpaired t-test. ^*^p<0.05, ^**^p<0.01.

**
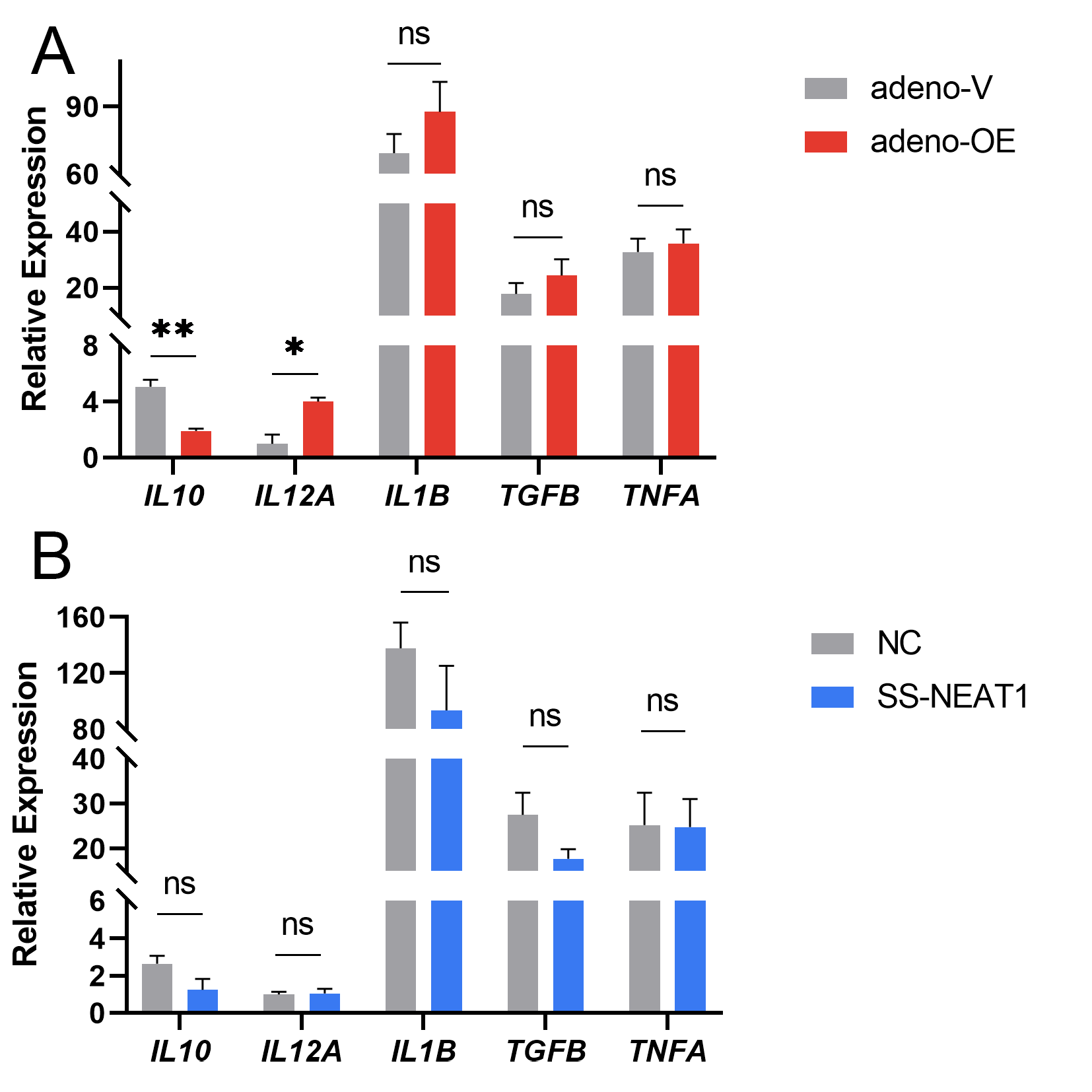
**

**Table S1.** Sequences of oligonucleotides

| Gene | Sequences (5’-3’) |
| --- | --- |
| *Neat1-*forward | TAAGGGTGTCATTGTGTTGTG |
| *Neat1-*reverse | AACTAGACCTGCCATTTCTCA |
| *IL6-*forward | TAGGACTGGAGATGTCTGAGG |
| *IL6*-reverse | TGTGGAGAAGGAGTTCATAGC |
| *TNFA-*forward | CAGGACTTGAGAAGACCTCAC |
| *TNFA-*reverse | GTCTGGAAACATCTGGAGAGA |
| *IL1B-*forward | TCTTCATTGCTCAAGTGTCTG |
| *IL1B-*reverse | TGCCACTGTAATAAGCCATC |
| *IL10-*forward | GTTTTCCCTGACCTCCCTCTA |
| *IL10-*reverse | GCTCCCTGGTTTCTCTTCCTA |
| *IL12A-*forward | TGTACCAGGTGGAGTTCAAGA |
| *IL12A-*reverse | CTCATCAATAACTGCCAGCAT |
| *TGFB-*forward | GAGCCCTGGACACCAACTATT |
| *TGFB-*reverse | TCCTTGCGGAAGTCAATGTAC |
| *ACTB-*forward | AAGGTGACAGCAGTCGGTT |
| *ACTB-*reverse | TGTGTGGACTTGGGAGAGG |
| *GAPDH-*forward | GGGAAGGTGAAGGTCGGAGT |
| *GAPDH-*reverse | GGGGTCATTGATGGCAACA |
| *hsa-miR-365a-3p-*hairpin primer | CTCAACTGGTGTCGTGGAGTCGGCAATTCAGTTGAGATAAGGA |
| *hsa-miR-365a-3p-*forward | TAATGCCCCTAAAAATCC |
| *hsa-miR-365a-3p-*reverse | CAACTGGTGTCGTGGA |
| *U6-*hairpin primer | AAAAATATGGAACGCTTCACG |
| *U6-*forward | GCTTCGGCAGCACATAT |
| *U6-*reverse | ATTTGCGTGTCATCCTTG |
| *Neat1-*siRNA targeted sequence | GCAGGTTGAAGGGAATTCT |
|  | GGACCACTTAAGACGAGAT |
|  | GCGAGGTGCCTTTACTACA |
| *Neat1-*Aso targeted sequence | GGGAGGGATGAGGGTGAAGA |
|  | GGGACAGACAGGGAGAGATG |
|  | AGGAGAAGGGAATGGTGGGT |

ASO: antisense oligonucleotides; siRNA: small interfering RNA.

**Table S2.** Clinical characteristics of included SLE patients.

| Characteristics | SLE(n=6) | HC(n=6) |
| --- | --- | --- |
| Sex, male/female(n) | 0/6 | 0/6 |
| Age (years) | 44.50±3.74 | 30.17±4.79 |
| Duration (months) | 2.83±0.79 |  |
| RBC (10^12) | 3.73±0.38 |  |
| WBC (10^9) | 4.06(3.14-10.54) |  |
| Hemoglobin (g/L) | 114.70±10.61 |  |
| Platelet count (10^9) | 183.00±25.74 |  |
| ESR (mm/h) | 19.33±6.15 |  |
| Urine protein, yes/no(n) | 3/3 |  |
| SLEDAI score | 9.67±2.88 |  |
| ANA>1:320, yes/no(n) | 6/0 |  |
| Anti-dsDNA (IU/ml) | 378.30±99.02 |  |
| Abnormal (low) complement C3, yes/no(n) | 3/3 |  |
| Abnormal (low) complement C4, yes/no(n) | 3/3 |  |
| Organ involvement, yes/no(n) | 4/2 |  |
| Glucocorticoids ^a^, yes/no(n) | 5/1 |  |
| Immunosuppressive drugs, yes/no(n) | 0/6 |  |

Data are presented as median (minimum-maximum) or mean ± SEM. ^a^: systemic usage of glucocorticoids within one year of enrollment. ANA: antinuclear antibody; SLE: systemic lupus erythematosus; SLEDA: systemic lupus erythematosus disease activity index; RBC: red blood cell; WBC: white blood cell; ESR: erythrocyte sedimentation rate.
